# Antibiotic prophylaxis at the time of dental implant placement: a cost-effectiveness analysis

**DOI:** 10.1186/s12913-022-08452-x

**Published:** 2022-08-22

**Authors:** Khrystyna Zhurakivska, Lucio Lo Russo, Lorenzo Lo Muzio, Vito Carlo Alberto Caponio, Luigi Laino, Claudia Arena, Nicola Cirillo, Giuseppe Troiano

**Affiliations:** 1 Department of Clinical and Experimental Medicine, University of Foggia, Via Rovelli 50, 71122 Foggia, Italy; 2 Department of Biomedical and Surgical and Biomedical Sciences Naples University, Naples, Italy; 3 Melbourne Dental School, The University of Melbourne, Carlton, VIC 3053 Australia

**Keywords:** Cost-effectiveness, Antibiotics, Antibiotic prophylaxis, Implants, Dental implant

## Abstract

**Background:**

Antibiotic prophylaxis during implant placement may improve implant short term survival. Nevertheless, use of antibiotics carries risks of adverse effects and antibiotic resistance. The aim of the present study is to compare the use of antibiotics in dental implant procedures in terms of costs and effectiveness.

**Methods:**

A decision-tree model was developed using TreeAge Pro Healthcare software. Two strategies were compared: *Antibiotics* and *No antibiotics* in implant placement procedures. The costs were calculated considering direct costs for implant placement, antibiotic costs, and costs for implant replacement in case of failure. Effectiveness was defined in terms of General Oral Health Assessment Index. Outcomes were evaluated as Incremental Cost Effectiveness Ratio (ICER). One-way sensitivity analysis and Probabilistic Sensitivity Analysis were performed for the most influential variables to test parameter uncertainty. Patient and healthcare perspectives were considered.

**Results:**

Antibiotic prophylaxis resulted to be cost-effective compared to no use of antibiotics (ICER = 14,692,64 and ICER = 3841,18, respectively for patient’s and healthcare perspective). The cost of antibiotics, cost of implant replacement in case of failure and probability of adverse effects significantly influenced the results.

**Conclusions:**

From an individual patient perspective, antibiotic strategy can be considered cost-effective, even when the cost of antibiotic therapy increases. We can conclude that the administration of antibiotics in association with implant placement is recommended in clinical practice, as it increases the success rate and makes the treatment more effective. However, attention should be placed when healthcare perspective is considered, particularly in terms of antibiotic resistance that may impact public health and associated costs.

## Background

Dental implant placement is a surgical procedure that aims to rehabilitate edentulous areas of the jaws [1]. Over the last decade, surgical methodology has been highly standardized, thus reaching an optimum level of predictability. However, in some cases, the process of osseointegration can be undermined by several factors, particularly intraoperative or postoperative infections [2, 3]. To avoid implant loss due to infection, antibiotic prophylaxis at the time implant placement has been advocated, and various molecules and regimens of administration have been proposed [4, 5]. However, the real effect of antibiotic use on implant survival and incidence of infectious complications is still unclear. A recent systematic review and meta-analysis addressed this issue by synthetizing scientific findings regarding the influence of antibiotic prophylaxis on implant early failure rate [6]. The authors showed that there is indeed a statistically significant improvement in patients treated by means of antibiotic therapy, compared to no use of antibiotics [6]. On the other hand, it is increasingly clear that antibiotics should not be prescribed indiscriminately due to their possible adverse effects and, above all, to the increasing worldwide problem of antibiotic resistance [7]. For these reasons, only procedures that really benefit from antibiotic treatment should be associated to their use. This dilemma makes it necessary to undertake a careful comparison of costs and benefits about the use of these class of drugs.

Cost-effectiveness analysis (CEA) is an analytic approach aiming at comparison of two or more alternative courses in terms of resource consumption and health outcomes. The result of such analysis is fundamental for an evidence-based decision making and an optimal allocation of health resources [8]. Costs and outcomes to consider can vary depending on the perspective of analysis. In dentistry, one of the following perspectives is usually used when conducting a CEA: society, third-party payer (insurance company or healthcare system), dental practitioner or patient population [9].

The purpose of the present study was to investigate whether the incremental cost of antibiotic prophylaxis during dental implants placement was justified by the consistent improvement in success rate of implant survival. Considering that the Italian public healthcare system does not cover dental procedures, which are paid by the patient to the private dental practitioner, our analysis was performed from the perspective of an individual patient. Furthermore, since antibiotic resistance may require additional use of healthcare resources, consideration of healthcare perspective was made and discussed as well.

To our best knowledge, this is the first study investigating the economic aspect of antibiotic administration during dental implant placement.

## Methods

This manuscript has been structured following the Consolidated Health Economic Evaluation Reporting Standards (CHEERS) [10]. The model compared the same clinical procedure, namely dental implant placement, performed with or without antibiotic prophylaxis (defined as the intake of antibiotics prior to or right after implant placement). Both patients and healthcare perspectives were analyzed by assessing incremental cost-effectiveness ratios (ICERs).

### Population

The model was set to reproduce a hypothetical situation in which a patient, in a good general health status, requires implant rehabilitation of a missing tooth. Specifically, the intervention that is being assessed in this study consists in the replacement of a posterior tooth (in which the aesthetic value of the rehabilitation may be considered negligible) in native bone, without any prior or contextual use of synthetic biomaterials.

Patients with comorbidities that require antibiotic prophylaxis (including individuals at risk of infective endocarditis and prosthetic joint infection, immunocompromised patients and those with certain metabolic disorders) were not considered in this analysis, since the prophylactic use of antibiotics is strongly recommended in these at risk patients [11] and is likely to influence their general health status.

The treatment was estimated to be performed in Italy by dentist in a private practice environment in 2021.

### Model structure

**Fig. 1:**
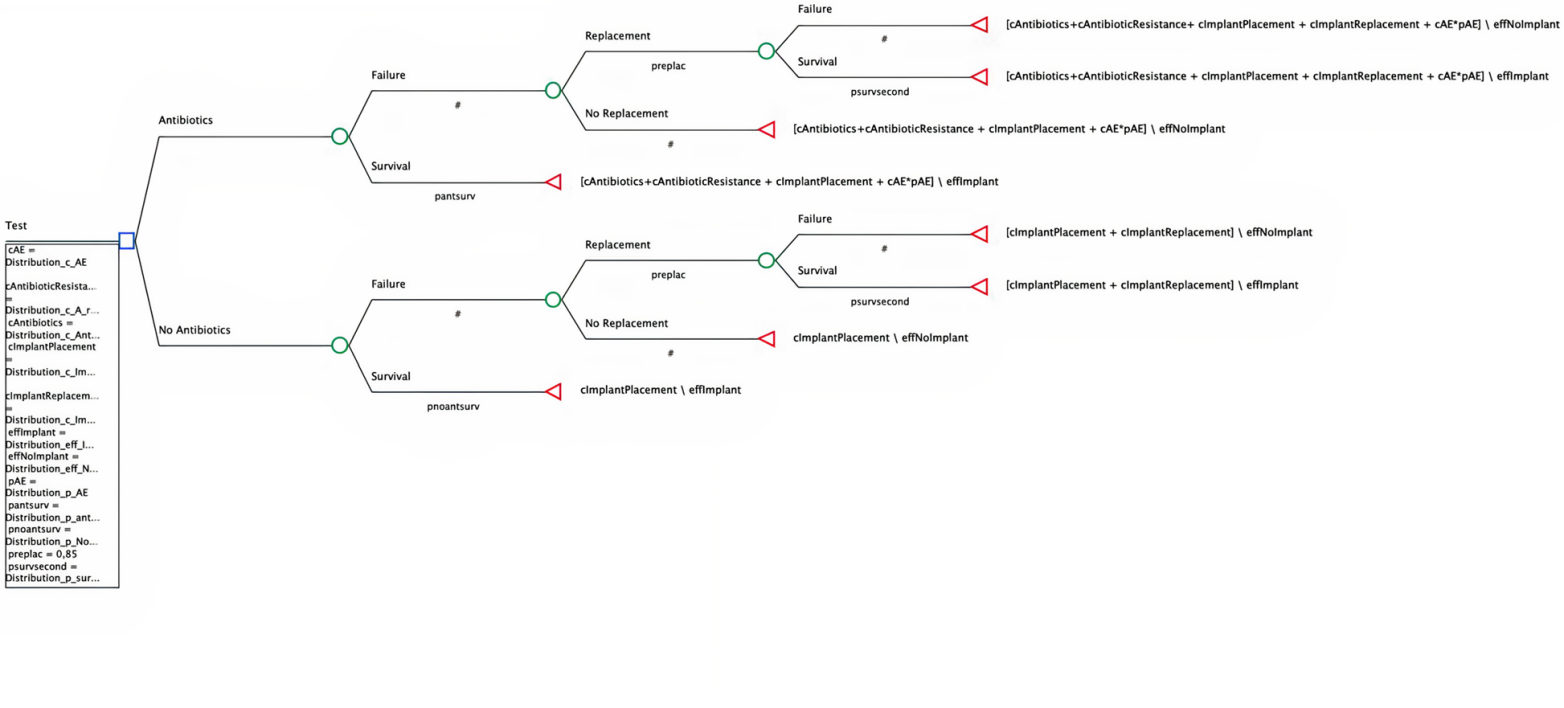
Model structure. The initial blue square box represents the decision node, from which two alternatives: Intervention (Antibiotics) and No intervention (No Antibiotics) branches depart. For each alternative two outcomes are possible: Failure or Survival, introduced by a chance node (green) and characterized by a certain probability. In case of Failure, another chance node has been placed introducing a potential Replacement of the implant with relative probabilities of Survival or Failure. Red triangles represent terminal nodes, where summary evaluations of payoffs, multiplied by probabilities, associated with each strategy of treatment are calculated

A decision tree with a short time horizon of 1 year (12 months) was built using TreeAge Pro Healthcare 2021 software (Fig.1). Such a time horizon was chosen since it was deemed long enough to capture relevant changes and success of implant placement and integration [12].

### Model assumptions

Personal characteristics such as age and sex were assumed not to influence the outcome, based on the results of available studies [4, 13–16]. An assumption was made based on authors judgement that the choice of implant replacement, in case of first implant failure, is made within the same year. No consideration about antibiotics use was made for implant replacement procedure, since the literature is lacking on this specific topic and the available data do not specify if the success rate of replaced implants is associated with the use of antibiotics [17].

### Data sources for model inputs and variables

#### Probabilities

Data regarding implant survival rates were obtained from a recently published systematic review and meta-analysis investigating the role of antibiotics in early implant failure, in which a Risk ratio of 0.31 has been reported for procedures associated with antibiotic prophylaxis compared to implant placement without antibiotic prophylaxis [6]. The studies included in this meta-analysis incorporate cohorts of patients from different countries who received multiple implant brands. No specific information is available about demographic data of the patients, except that they were in good health status.

The probability of implant replacement in case of failure was assumed to be of 85%, with a survival rate of 89%, according to Troiano et al. [17] in case of immediate implant replacement.

Regarding antibiotic prophylaxis, the cost of single course Amoxicillin taken pre-, peri- or post-operatively [17] was considered in terms of direct costs and costs of adverse effects (AE). The most common AE were considered: allergy, cutaneous rushes, gastrointestinal mild to severe manifestations, anaphylaxis. The probability of these non-fatal adverse effects of single course was set at 0.000023, and probability of fatal AE was set at 0.00, according to Thornhill et al. [18].

#### Costs

Costs of implant placement were obtained from database sourced by *statista.com* [19] reporting average costs of dental implant in private practice in Italy referred to 2018.

The cost of single course of Amoxicillin was estimated in €10.20, based on average dispensing costs [20].

The costs of AE management were estimated considering medication prescription for mild-moderate symptoms (antihistamine or antidiarrheal) and hospitalization costs for anaphylaxis and severe gastrointestinal AE. The direct cost of antihistamine/antidiarrheal preparations was assumed to be of 10.00€ per 2 days treatment [21, 22]. In case of severe AEs, hospitalization costs are assumed to be fully covered by the national health system (Italy). So, when healthcare payer’s perspective was considered, Diagnosis-related group (DRG) codes were used to estimate the costs of hospitalization. In particular, DRG 447 code was used for allergic reactions treatment, with a cost of € 1404.00 reported by official Italian government DRG document [23].

A separate consideration was made to estimate the cost of antibiotic resistance. The hidden societal costs of antibiotic resistance were quantified to be equivalent to $13 for each ambulatory antibiotic prescription in the United States [24]. Other researchers estimated the cumulative cost of antimicrobial resistance to be of 9,3$ per single course of broad-spectrum penicillin consumed in the United States [25] In this study, both direct and indirect costs were considered in the monetary evaluation of antibiotic resistance [25]. An average value of 11,15$ was chosen for the analysis. The amounts were converted to EUR, based on exchange rate of 2021, at USD1 = EUR0.85.

All the costs were adjusted for inflation to 2021 (1,8%) and were all annual costs. Inflation rates were obtained from Eurostat database (https://ec.europa.eu/eurostat/).

#### Effectiveness

The effectiveness of each strategy was valued based on data available in the scientific literature. In particular, we used the General Oral Health Assessment Index (GOHAI), as detailed in the study of Korenori et al. [26]. GOHAI is a specific oral health assessment index that expresses the QOL related to oral cavity. GOHAI comprises three physical areas: eating, swallowing and pronunciation and two psychosocial aspects, consisting of aesthetic appreciation and sociability. Furthermore, pain and discomfort are evaluated through the investigation of medicines use and hypersensitivity evaluation. The total score is expressed in the range of 12–60, but for our purposes the GOHAI value was available in the continuous values between 0 and 1 based on beta distribution (0: no satisfaction; 1: maximum satisfaction). The established effectiveness were 0.88 for implant rehabilitation and 0.71 for implant loss, as reported by Korenori et al. [26].

Considering a time horizon of 1 year, the above-mentioned utilities were used for qualitative effectiveness evaluation and no quantitative calculation of Quality-adjusted life years was made.

Variables were defined for each parameter considered in the analysis. Input values assigned to the variables, their ranges and sources are summarized in Table 1.

**Table 1:** Description of variables, values, sources and tested ranges

Variables	Description	Root Definition	Source	Costs adjusted for inflation	Low	High
cAE	cost Adverse effects of antibiotics	705	DRG, Health ministery of Italy [23] (2013)	708.6	10	1800
cAntibiotics	Costs of antibiotics	10.20	Assumed based on dispensing costs 2021, reported by AIFA [20].	10.20	5	100
cAntibioticResistance^a^	Cost of antibiotic resistance	9.47	Michaelidis et al.; Shrestha et al. [24, 25]	13.97	2.9	32.16
cImplantPlacement	Cost of Implant Placement	1002	Statista.com [19] (2018)	1002	500	1500
cImplantReplacement	Cost of Implant Replacement	1102	Assumed based on Cost of Implant Placement	1102	0	1600
effImplant	Effectiveness Implant Placement	0.88	Korenori et al. [26] (2018)		0,5	0,9
effNoImplant	Effectiveness Lost implant	0.71	Korenori et al. [26] (2018)		0,3	0,8
pAE	probability Adverse effects antibiotics	0.000023	Thornhill et al. [18] (2015)		0,00001	0,05
pantsurv	Probability of survival with antibiotics	0.9878	Canullo et al. [6] (2020)		0,6	0,9878
pnoantsurv	Probability of survival without antibiotics	0.9567	Canullo et al. [6] (2020)		0,6	0,9567
preplac	Probability of implant replacement	0.85	Assumed		0,3	0,9
psurvsecond	Probability of survival after secondary placement	0.89	Troiano et al. [17] (2021)		0,6	0,89

^a^The cost of antibiotic resistance was considered only for societal perspective

#### Sensitivity analysis

Probabilistic sensitivity analysis (PSA) was performed using Monte-Carlo simulation repeated 1000 times and including the following variables with their distributions: cost of antibiotics, cost of antibiotic resistance, cost of adverse effects, cost of implant placement, cost of implant replacement, effectiveness of implant survival, effectiveness of implant loss. Normal distribution was set for cost variables and beta distribution was selected for effectiveness. Acceptability curves were generated for WTP values ranging between 0 and 10,000€.

An influence analysis was performed to rank variables in order of their influence on the magnitude of the model output. Subsequently, one-way sensitivity analysis was performed for the most influential variables to test the parameters uncertainty. The tested ranges were decided based on expert’s opinion and are listed in Table 1.

For sensitivity analysis, WTP threshold was set at 3000€ for both perspectives, based on data reported in studies investigating patients’ preferences for implant rehabilitation of single-tooth gap in Italy [27].

## Results

Implant placement with concurrent use of antibiotics was cost-effective compared to the same procedure without antibiotics. Specifically, it was both less expensive (1023.64€ and 1037.61€ from patient’s and healthcare perspective, respectively Vs 1042.56€ for no antibiotic approach) and slightly more effective (0.87949 vs 0.87821 GOHAI), when compared to No Antibiotic approach with an Incremental Cost-Effectiveness Ratio of 14,692,64 for patient’s and 3841,18 for healthcare perspectives. The costs, effectiveness, ICERs and NMBs resulted from examination of both perspectives are reported in Table 2. The only difference between patient and healthcare/societal perspectives is represented by the cost of Antibiotic resistance, which in Italy falls on healthcare provider that is covered by national health system (healthcare cost).

**Table 2:** Incremental costs and effectiveness for each strategy

Strategy	Cost (EUR)	Incremental Cost	Effectiveness (GOHAI)	Incremental E	ICER	NMB	
Societal perspective							
All referencing common baseline							
Antibiotics	1037,61		0,879,495			1600,87	undominated abs. Dominated
No Antibiotics	1042, 56	4,95	0,878,208	−0,00129	−3841,18	1592,06	
Patient’s perspective							
All referencing common baseline							
Antibiotics	1023,64		0,879,495			1614,84	undominated abs. Dominated
No Antibiotics	1042,56	18,92	0,878,208	−0,00129	−14,692,64	1592,06	

*C* Costs, *E* Effectiveness, *abs* Absolutely, *NMB* Net Monetary Benefit, *ICER* Incremental Cost-Effectiveness Ratio

**Fig. 2:**
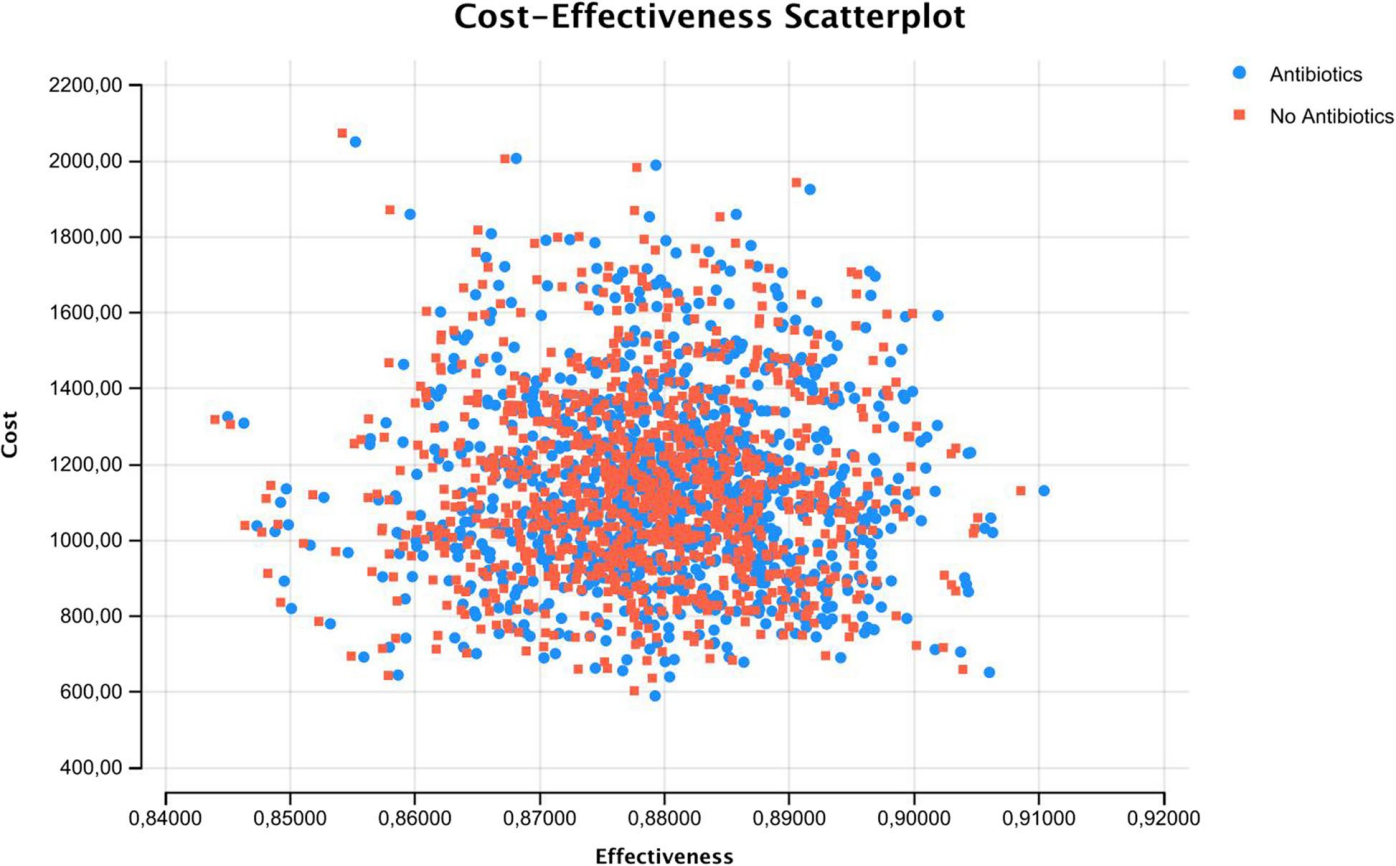
CE Scatterplot (Monte Carlo Simulation)

**Fig. 3:**
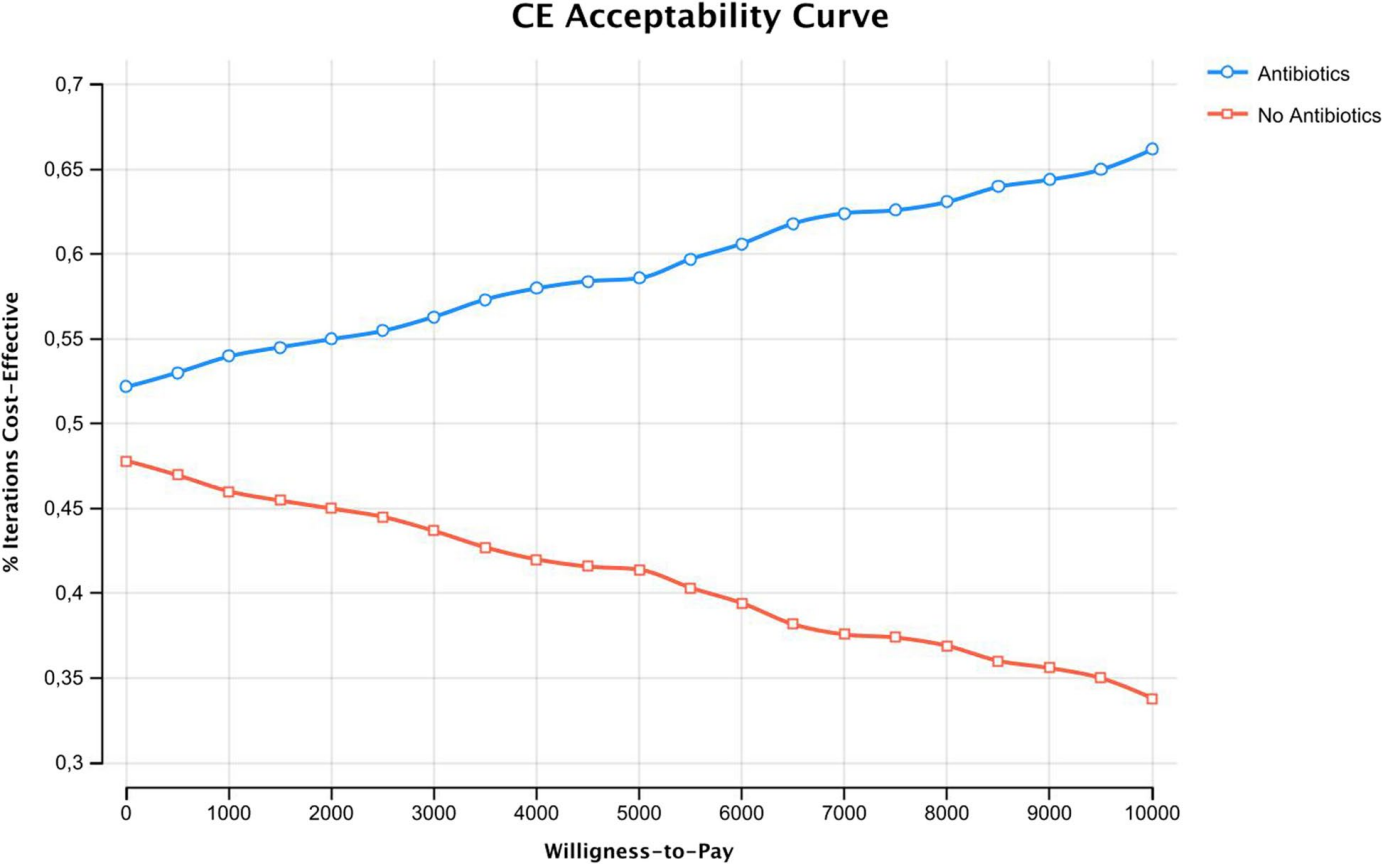
Cost-effectiveness Acceptability curves

**Fig. 4:**
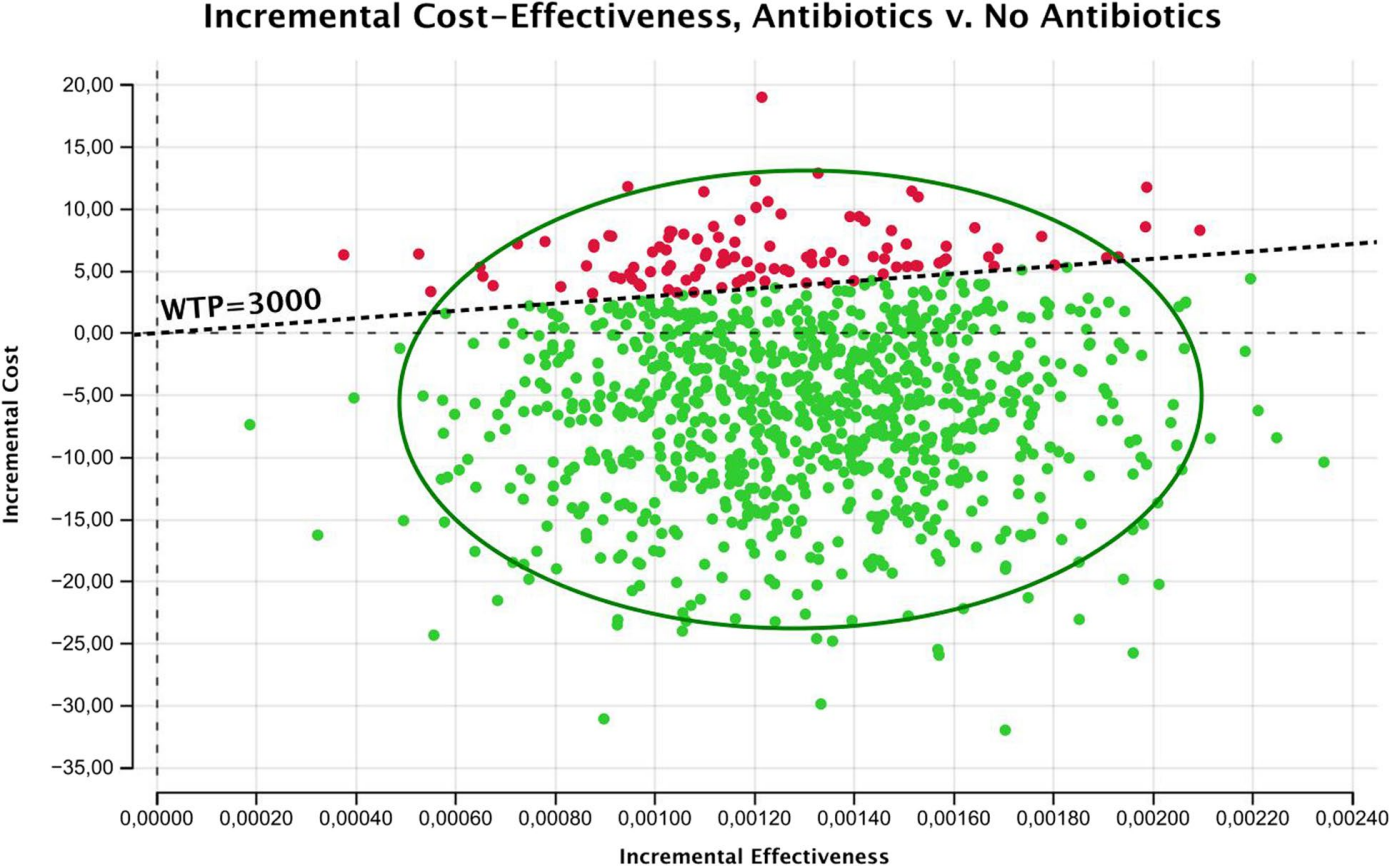
ICE Scatterplot (Monte Carlo Simulation)

PSA performed with 1000 Monte Carlo simulations revealed that the points representing CE of the strategies are widely distributed on the plane, with no clear prevalence of one strategy on another (Fig. 2). Acceptability curves analysis performed for a range of WTP between 0 and 10,000 confirmed that the Antibiotic strategy remains dominant for each value (Fig. 3). Incremental cost-effectiveness scatterplot with a WTP threshold of 3000,00 € shows the great frequency (758/1000) of iterations in which Antibiotic strategy results to be more dominant (Fig. 4).

Sensitivity analysis performed from healthcare perspective showed relevance of three variables: cost of antibiotics, cost of implant replacement and probability of adverse effects for dominance persistence. In particular, cost of antibiotics, probability of adverse effects and cost of antibiotic resistance are inversely proportional with Antibiotic strategy dominance and at hypothetical thresholds of 18,59€ for the cost of Antibiotics, a probability of adverse effects greater than 0,01, and a cost of antibiotic resistance greater than 22,36€ the two strategies become neutral for a WTP of 3000€.

Conversely, the cost of implant replacement is directly correlated with Antibiotic strategy dominance, since a high price of Implant replacement encourages Antibiotic administration to avoid implant failure. In particular, for a cost of Implant replacement higher than 754,04€, the Net Monetary Benefit of Antibiotic strategy becomes positive.

**Fig. 5:**
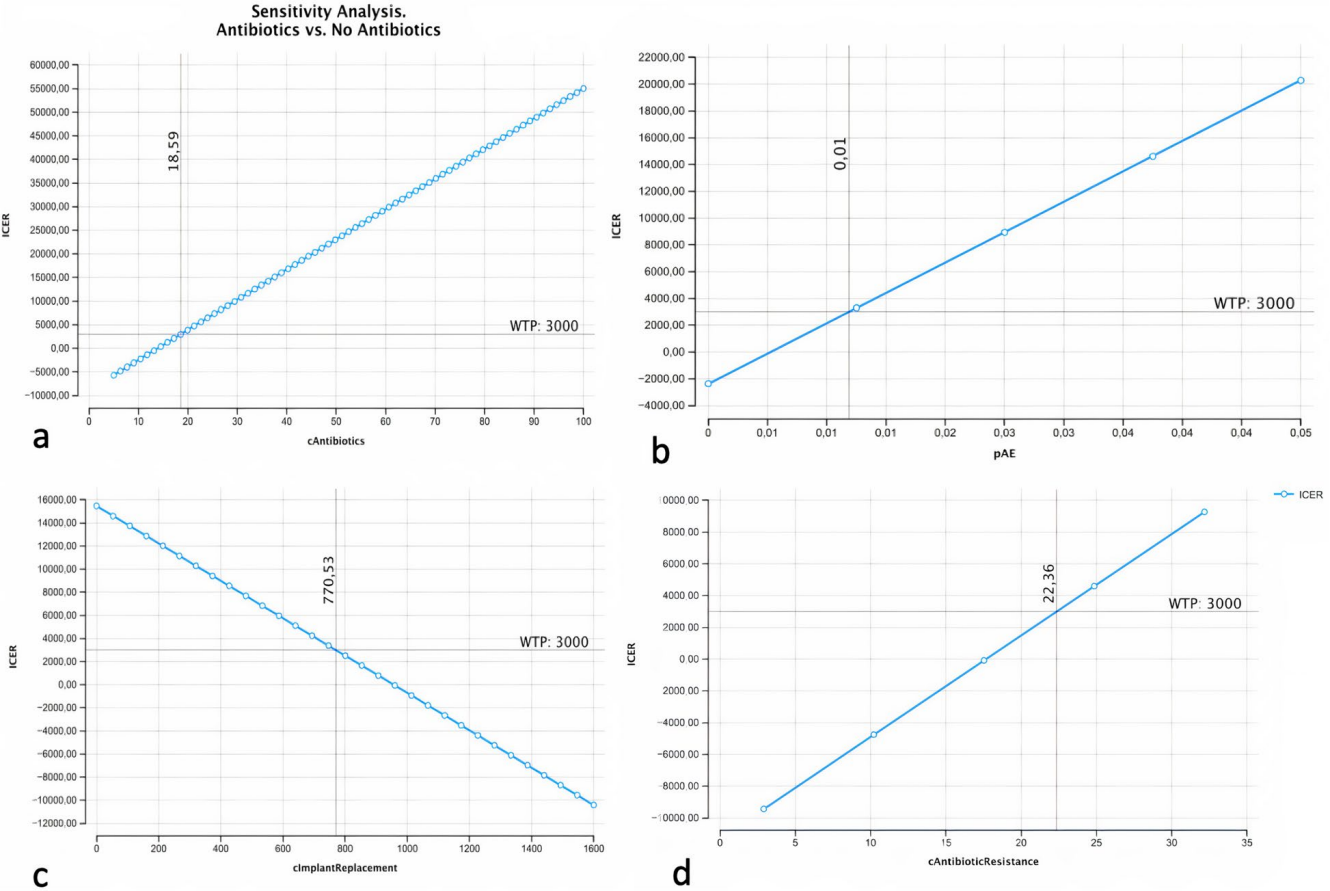
One-way sensitivity analysis of the most relevant variables. **a** cost of antibiotics (cAntibiotics); **b** probability of adverse effects (pAE); **c** cost of implant replacement (cImplantReplacement); **d** cost of Antibiotic resistance (cAntibioticResistance)

The results of one-way sensitivity analysis for the most relevant variables are graphically represented in Fig. 5 and threshold reports of sensible variables are shown in Table 3.

**Table 3:** Threshold values of sensible variables

Variable	Value	Tested range (min-max)	Threshold
**cAntibiotics**	24.85	5–100	20,15
**cImplant_replacement**	1101	0–1600	770,53
**pAE**	0.000023	0,00001-0,05	0,01

*cAntibiotics* Cost of antibiotics, *cImplant_replacement* Cost of implant replacement, *pAE* Probability of adverse events

**Fig. 6:**
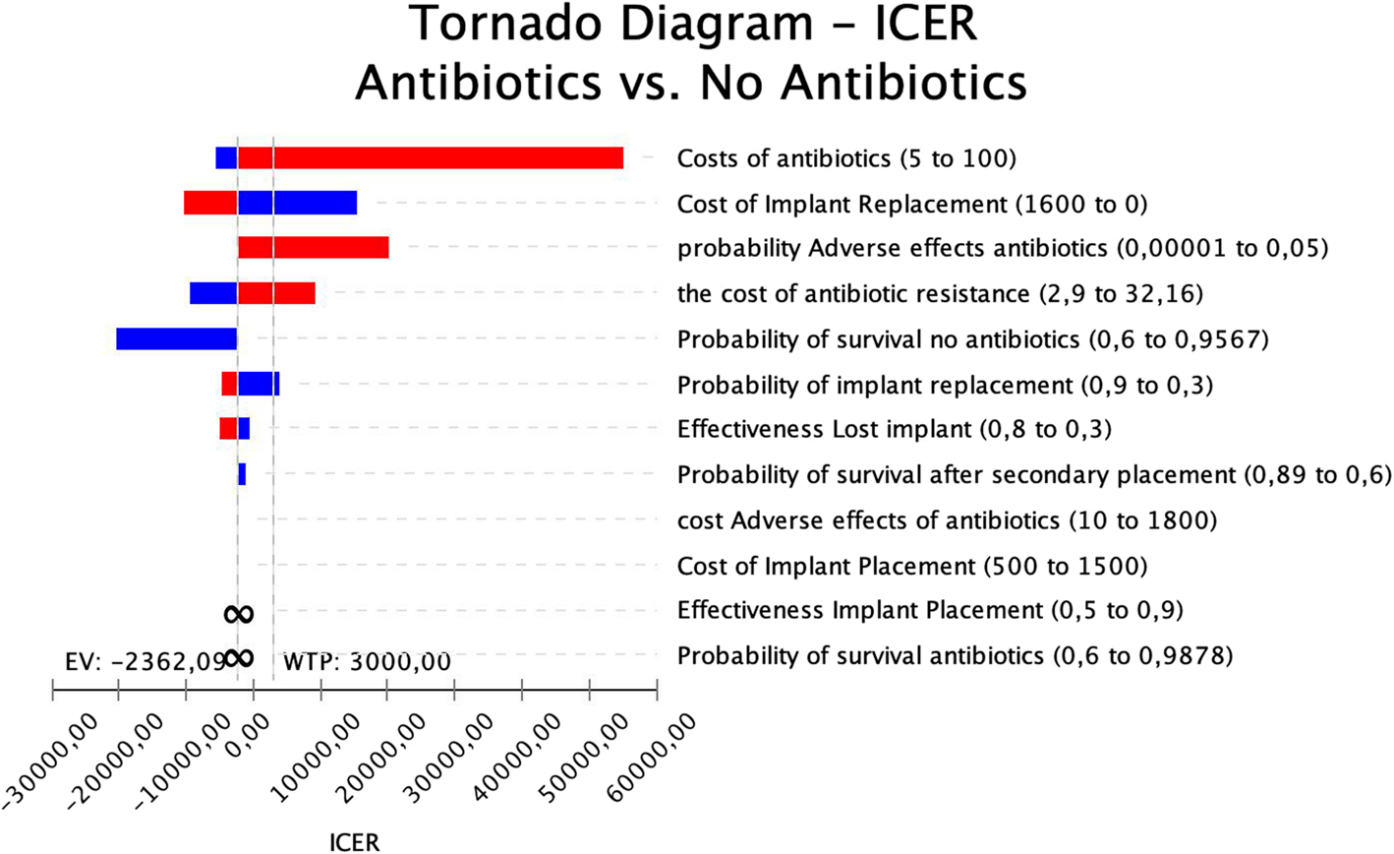
Tornado diagram. Tested variables are ranked in order of their overall influence on the magnitude of the model output

Variables that mostly influenced the relative cost-effectiveness of the strategy were: Costs of Antibiotics, Cost of Implant replacement, Probability of Adverse effects, Probability of survival without antibiotic prophylaxis and probability of Implant replacement. The ranking of variable influencing ICER is graphically represented in the Tornado diagram (Fig. 6).

## Discussion

Currently, antibiotic prophylaxis is recommended in dental practice only in some specific conditions, including treatment of acute odontogenic and non-odontogenic infections and prevention of local and focal infections in susceptible patients during invasive procedures [11]. Unfortunately, there is often an abuse in the prescription of antibiotics by practicing dentists, who neglect the risk of adverse effects and antibiotic resistance [28]. Among dental interventions for which antibiotic prophylaxis is often suggested there is implant surgery. In the present study, we compared dental implant placement performed with or without antibiotic prophylaxis.

The prescription of antibiotics in dental implantology has been commonly justified by the fact that implant insertion is an invasive surgical procedure that is performed in an infected environment (oral cavity) and involves the insertion of a foreign body for which an integration with the surrounding tissues is expected. A recent meta-analysis confirmed a slightly greater short-term success of implant rehabilitation in patients undergoing antibiotic prophylaxis, compared to patients who did not take antibiotics during implant placement [6]. However, this conclusion refers only to the reduction of implant failures and does not take into account other aspects associated with the use of antibiotics. A recent article estimated that the total societal cost of antibiotic resistance attributable to each ambulatory antibiotic prescription in US is roughly $13 [24]. Another study estimated the economic costs of antimicrobial resistance in Thailand and United States, based on type of responsible pathogen and antibiotic class driving resistance [25], and were assumed to be valid for both the low/middle and high-income countries. The costs associated with antimicrobial resistance were considered from several points of view, such as patient, healthcare and societal perspectives. The results demonstrated that antibiotic resistance is responsible for increase of morbidity and mortality, loss of earnings, higher toxicity of 2nd line drugs, longer hospital admissions, and costs incurred for development of alternative drugs. For our analysis, we considered the resistance costs estimated for broad spectrum penicillin in US. Nevertheless, the complexity of such an estimation due to variation in populations, drugs, healthcare system and societal organization should be considered, and an appropriate sensitivity analysis should always be undertaken to verify the robustness of the model.

The purpose of our analysis was to integrate biological and economic costs and benefits deriving from the use of antibiotics in patients undergoing implant surgery and to compare these with a scenario where no antibiotics are used. The time horizon for decision analysis was set at 1 year, since it was deemed long enough to capture relevant changes and outcomes of implant therapy. The sources of the parameters modelled in our study were various: meta-analyses, original studies, electronic databases. Because of the variable robustness of each source, uncertain data and assumptions were then addressed in the sensitivity analysis. In terms of perspective, we predominantly evaluated patients’ perspective given that dental procedures are mainly private in Italy and the costs are incurred directly by patients. Nevertheless, since some costs deriving from the use of antibiotics (e.g. severe adverse effects management, consequences of antibiotic resistance) may involve the public health system and the wider society, a healthcare perspective has also been considered in the present study. Both perspectives revealed a dominance of antibiotic prophylaxis, as it resulted to be both cheaper and more effective (ICER equal to 3292,44 and 14,672,1€) for both, healthcare and patients perspectives. Productivity loss was not considered in costs evaluation, since implant placement is an outpatient intervention that usually takes place in a few hours and does not require hospital stays. Therefore, we assumed that implant placement did not affect patients’ work or personal life significantly. We can reasonably speculate that if productivity loss had been considered, it would have increased the convenience of the antibiotic strategy even further, as it would have reduced the possibility of having to undergo implant replacement in case of implant failure. The most difficult parameter to determine was the QOL related to implant rehabilitation, since available data regarding oral health related QOL are variable [29]. GOHAI was used as the indicator of effectiveness concerning oral health QOL, as suggested by some studies [26, 30]. Values considered for assessment of efficacy (0.88 and 0.71 for implant rehabilitation and lost implant, respectively) refer to a specific situation of single molar implant rehabilitation. In case of anterior implant placement, the utility measure can vary significantly since aesthetic factors should be considered. Even in this case, if aesthetic factors were considered, the importance of implant success would likely increase, and therefore antibiotic therapy would be even more cost-effective. GOHAI is a widely used index for oral health-related quality of life (OHRQOL) evaluation. It was chosen for utility measurement because deemed to be sensitive enough to capture small changes in health status, such as those determined by implant rehabilitation of one tooth. We are aware that this is score may not be interpreted as a suitable weight for the construction of Quality-adjusted life-years. Nevertheless, since the time horizon used in our study was 1 year, the utility values could be used for effectiveness measurement.

Sensitivity analysis revealed that cost of antibiotics, cost of implant replacement in case of failure, and probability of adverse effects are the most relevant factors in determining the dominant strategy. In particular, if the cost of antibiotics (including the estimated cost of antibiotic resistance) exceeded €20.15, antibiotic prophylaxis would lose its convenience in terms of ICER. Although this threshold value is much higher compared with the dispensing costs of antibiotics (about €10), it could still be exceeded if the healthcare cost was considered. The same inversion of convenience would happen if the probability of adverse effects was significantly higher (> 0.01) or if the cost of implant replacement was significantly lower (< €754,04).

Regarding the willingness to pay, the threshold of 3000€ was estimated to be valid for both patient and societal perspectives. From a societal perspective, it is difficult to estimate what is the benefit of single-tooth replacement and how much additional taxes patients/taxpayers are available to pay to support public dental treatment and all associated costs. Nevertheless, in our case the antibiotic strategy resulted to be both less expensive and more effective, so dominant even if the WTP was 0 €.

The main limitations of the study are related to a close connection with the specific context represented by Italian healthcare organization. This aspect limits the generalizability of the results and conclusions. Nevertheless, the consideration of a wider healthcare perspective allowed us to expand the possible applicability of our model. The second limitation of the study concerns the source of certain values used for defining the variables of interest. In particular, effectiveness estimation and some probabilities were obtained from individual original studies. Conversely, other measures were derived from meta-analyses, and therefore the values were more reliable. This issue was determined by the fact that few data are available for such a specific condition like single-tooth replacement by implant. Furthermore, the utility evaluation was obtained from an article evaluating OHRQOL in the Japanese population. This setting may be slightly different for the Italian population, even though no big differences in terms of health status exist between Japan and Italy [31]. To address this issue, wide ranges of values were used in the sensitivity analysis in order to test the uncertainty of the values.

## Conclusions

The use of antibiotics during implant placement resulted to be a cost-effective strategy, compared with no use of antibiotics. However, the dominance of the strategy involving the use of antibiotics is sensitive to the cost of antibiotics and to the cost of a possible implant replacement. From single patient’s perspective, antibiotic strategy can be considered cost-effective even when the cost of antibiotic therapy increases. However, attention should be placed when healthcare impact is considered, since the costs of management of antibiotic resistance incurred by the healthcare system could potentially undermine the certainty of the choice.

## Data Availability

“All the data are available from the corresponding author after reasonable request”.

## Abbreviations

CEA: Cost-effectiveness analysis
ICERs: Incremental cost-effectiveness ratios
AE: Adverse effects
DRG: Diagnosis-related group
GOHAI: General Oral Health Assessment Index
ISTAT: Italian National Institute of Statistics
QOL: Quality-of-life
WTP: Willingness to pay
